# Liquid-Phase-Assisted Catalytic Nitridation of Silicon and In Situ Growth of α-Si_3_N_4_

**DOI:** 10.3390/ma15176074

**Published:** 2022-09-01

**Authors:** Zhenglong Liu, Zhinan Chai, Chao Yu, Jun Ding, Chengji Deng, Xiangcheng Li, Hongxi Zhu

**Affiliations:** 1 The State Key Laboratory of Refractories and Metallurgy, Wuhan University of Science and Technology, Wuhan 430081, China; 2 Guangzhou Newlife New Material Co., Ltd., Guangzhou 511356, China

**Keywords:** liquid-phase-assisted, catalytic nitridation, α-Si_3_N_4_, microstructure

## Abstract

Si_3_N_4_ powders were synthesized with Fe, Co, or Ni as catalysts using Si powder at 1250 °C in a nitrogen atmosphere by liquid-phase-assisted catalytic nitridation synthesis (LPA–CNS). The catalytic effects of the metals on the nitridation of silicon powder were investigated by mixing the powder with 2 wt% by mass of Fe, Co, or Ni in a high-temperature liquid phase in flowing nitrogen. The α-Si_3_N_4_ micro-morphology could be effectively changed by adjusting the type of catalyst in the initial reaction mixtures. Fe, Co, and Ni promoted the formation of α-Si_3_N_4_ at 1250 °C and controlled the morphology of the α-Si_3_N_4_ particles. The hexagonal flakes of α-Si_3_N_4_ with a better defined morphology were obtained using Ni as the catalyst, compared to that obtained from the other two catalysts.

## 1. Introduction

Si_3_N_4_-based ceramics have been widely used in steel, aerospace, chemical, and electronic fields due to their strong oxidation resistance, stable chemical properties, and excellent mechanical properties [1,2,3,4,5]. In general, α-Si_3_N_4_ powder is preferred to β-Si_3_N_4_ powder as a raw material for fabricating compacted bodies because the α-Si_3_N_4_ starting powder has a higher reactivity that easily forms elongated Si_3_N_4_ grain morphology, which has excellent mechanical properties [4,5]. Due to these appealing properties, Si_3_N_4_ has been widely studied and has become the most promising structural material, especially for high temperature applications [6,7]. However, the high cost of fine Si_3_N_4_ powders is the prime obstacle to the regular application of Si_3_N_4_-based materials. Therefore, the fabrication of α-Si_3_N_4_ powders with tailored properties through a cost-effective synthesis method is a research topic of great interest.

At present, the preparation methods of silicon nitride are mainly divided into organic preparation methods and inorganic preparation methods. The organic method is mainly used to synthesize precursors and to prepare silicon nitride through the pyrolysis of the precursors [8,9,10]. Some inorganic preparation methods have been adopted to prepare silicon nitride powder, including the nitridation of Si powder [11], carbothermal reduction nitridation [12,13], and combustion synthesis [14,15]. However, these techniques still suffer various disadvantages, such as a broad particle size distribution, difficulty in size and composition control, powder reunion, high nitridation temperature, and long holding time. High temperature liquid phase synthesis method is an environmentally friendly technology, widely applied in the preparation of powder [16,17,18,19], nano coating [20,21,22,23], and porous materials [24,25,26]. However, there are only a few available reports on the high temperature liquid phase synthesis of silicon nitride powder.

It was well known that transition elements can regulate the kinetics of Si nitridation and accelerate the growth of α-Si_3_N_4_ [27,28]. It should be noted that, although it has been reported that metals, metal oxides, metal nitrates, or metal nanoparticles were used as a catalyst for the accelerated nitridation of Si powder [29,30,31,32,33,34], most of the previous studies have focused on the limited choices of catalysts and complex synthesis methods. In addition, the enhancement of α-Si_3_N_4_ formation is strictly limited to the presence of molten alloy liquid phases [34,35] but most catalysts do not play a distinctly accelerated role in the information of α-Si_3_N_4_ powder. Other research groups have made some attempts on controllable preparation of α-Si_3_N_4_ [36,37] including our previous contribution [38,39,40]. However, the influence of different transition metal catalysts on the fabrication of α-Si_3_N_4_ powder is still unclear.

In this study, the synthesis of Si_3_N_4_ powder with different microstructures from by liquid-phase-assisted catalytic nitridation synthesis (LPA–CNS) in the NaCl–NaF molten salt system. The nitridation processes were carried out simultaneously with the three metal catalysts under identical reaction conditions, such as nitridation temperature, nitridation time and catalyst amount.

## 2. Materials and Methods

**Figure 1:**
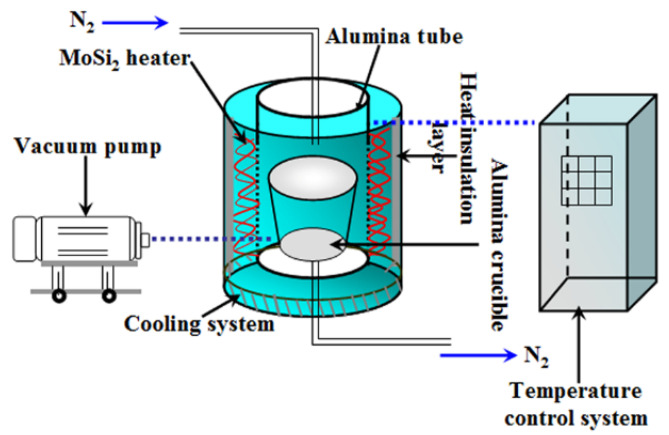
Nitridation apparatus for the preparation of α-Si_3_N_4_ powder.

The details of the synthesis process are described as follows: 35 wt% Si powder (99% purity, grain size 9 μm, Beijing Xing Rong Yuan Technology Co. Ltd., Beijing, China); 65 wt% analytical grade salt mixture composed of 95 wt% sodium chloride (NaCl, 99% purity, Sinopharm Chemical Reagent Co., Ltd., Shanghai, China) and 5 wt% sodium fluoride (NaF, 99% purity, Sinopharm Chemical Reagent Co., Ltd., Shanghai, China); and 2 wt% Fe, Co, or Ni powder (99.99% purity, grain size 2 μm, Beijing Xing Rong Yuan Technology Co., Ltd., Beijing, China) were dry-mixed in an alumina mortar for 1 h. Then, five grams of the mixed powder containing Si and Na salts and the catalyst were placed in an alumina crucible. The nitridation of the silicon powder was carried out in a vertical alumina tube furnace, which is shown as a diagram in Figure 1 together with the gas supply system and vacuum system. Since the reaction between N_2_ and Si is highly exothermic, the samples were initially heated at 1150 °C for 1 h in the alumina-tube furnace. The reaction temperature was then raised to 1250 °C and the reaction was continued for 5 h under a nitrogen atmosphere. After cooling to 25 °C under nitrogen, the as-obtained solidified mass was repeatedly washed with hot distilled water and filtered to remove the residual salts. Finally, the obtained silicon nitride paste, prior to its characterization, was dried in oven at 110 °C for 12 h.

The product was collected, and samples were prepared accordingly for various characterization techniques. The phase composition of the as-prepared samples was identified by X-ray diffraction (XRD, X’Pert Pro, Philips, Eindhoven, The Netherlands) analysis. The spectra from 10° to 80° (2θ) were recorded using Cu Kα radiation (λ = 0.154 nm) at 40 mA and 40 kV, at a scan rate of 2°/min and a step size of 0.03°. ICSD cards No. 035561, 074749 and 043403 were cited, respectively. The infrared (IR) spectra of the samples were recorded on a Fourier transform infrared (FTIR, NICOLET iS50, Thermo Fisher Scientific, Waltham, MA, USA) spectrometer in the range 300–4000 cm^−1^. The microstructure of the samples was characterized using a field-emission scanning electron microscope (FESEM, Nova nano 400, FEI, Waltham, MA, USA) and a transmission electron microscope (TEM, JEM2100, JEOL, Tokyo, Japan). Samples for SEM analysis were coated with gold. Samples for TEM analysis were dispersed in absolute ethanol by ultrasonication, and a drop of the suspension was placed on a copper grid and air-dried. Selected-area electron diffraction (SAED) along with energy dispersive spectroscopy (EDS) was used to identify the phase composition of the samples. Finally, the property of powder was characterized by Brunauer–Emmett–Teller (BET, Autosorb-1-MP/LP, Quantachrome, Boynton Beach, FL, USA).

## 3. Results

**Figure 2:**
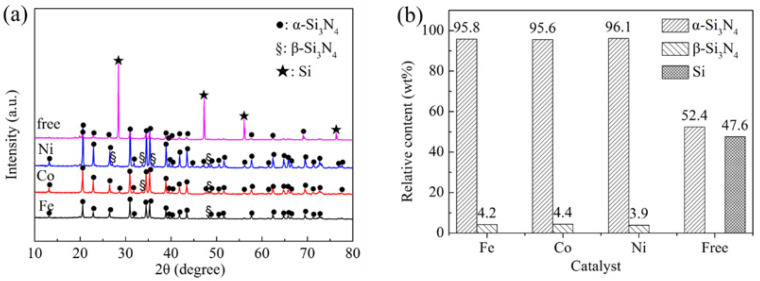
XRD patterns of samples and relative content of the crystalline phases in the final products obtained by 5 h of nitridation at 1250 °C using various catalysts. (**a**) XRD patterns and (**b**) relative content.

A series of experiments were performed in which the type of catalyst was varied. There are amounts of unreacted Si and a small amount of α-Si_3_N_4_ in the catalyst-free sample, which indicates that only a small amount of Si has been nitrided, as shown in Figure 2a. However, it can be clearly seen that the residual Si peaks (corresponding to (111), (220), (311) and (331)) distinctly decreased or disappeared. Meanwhile, both α-Si_3_N_4_ and β-Si_3_N_4_ were identified in the samples containing catalyst, indicating that Si powder should be nitrided completely under the effects of Fe, Co, and Ni catalysts. Moreover, Ni catalysts give a higher intensity of α-Si_3_N_4_ phase when compared with using Co and Fe, indicating that the amount of α-Si_3_N_4_ in the samples containing Ni is more than those in samples containing Co or Fe. The relative contents of crystalline phases in the samples with various catalysts after 5 h nitridation at 1250 °C were calculated by the Rietveld refinement method, and the results are shown in Figure 2b. The addition of transition metal catalyst in Si powder led to a higher content of α-Si_3_N_4_ phase than that obtained from the catalyst-free sample. Only 52.4 wt% α-Si_3_N_4_ was formed in the catalyst-free samples. On the contrary, the samples containing the Fe, Co, and Ni catalysts demonstrated over 95.6% α-Si_3_N_4_ and less than 4.4% β-Si_3_N_4_ in the final product. Therefore, adding these transition metals into silicon raw material is a promising choice for the quality improvement of the α-Si_3_N_4_ produced by the high temperature liquid phase synthesis method.

**Figure 3:**
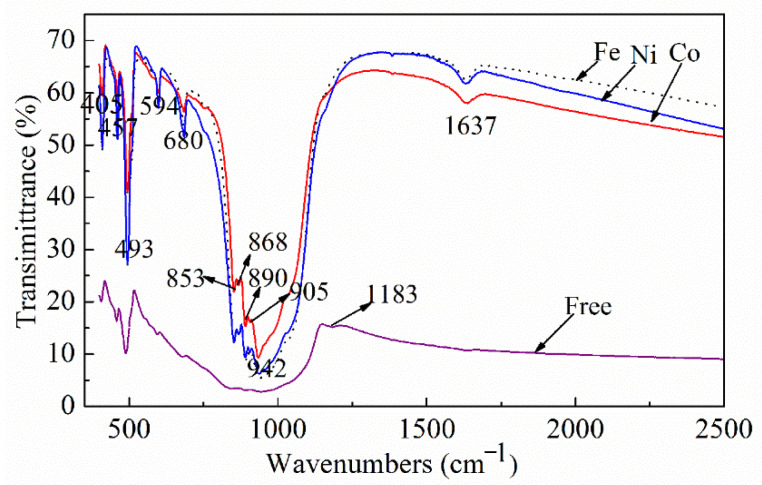
IR spectra of silicon nitride in the samples obtained using various catalysts and the catalyst-free sample.

The as-prepared α-Si_3_N_4_ samples were further characterized by FTIR. The FTIR spectra of the sample in Figure 3 show two absorption bands, one at 800–1100 cm^−1^ with a peak maximum at ~942 cm^−1^ and the other at 400–600 cm^−1^ with a peak maximum at ~493 cm^−1^, which correspond to the Si-N-Si asymmetric and symmetric stretching modes, respectively [41]. The bands at 942, 890, 853, 680, 594, 493, 457, and 405 cm^−1^ indicate the distinct absorptions of α-Si_3_N_4_ [42]. These bands become sharper in the spectra of the samples with the added catalysts, indicating the formation of a well-defined crystal structure of the α-Si_3_N_4_ phase. The peak at 1183 cm^−1^ corresponds to the stretching vibration of Si–Si bonds owing to unreacted silicon in the catalyst-free sample [43]. The peak at 1637 cm^−1^ is attributed to the water absorbed during the analysis [44]. The superimposed IR spectrum of α-Si_3_N_4_ further confirms the formation of α-Si_3_N_4_ as the primary phase in the samples, corroborating with the XRD results. In addition, it confirms the presence of residual Si in the catalyst-free sample, as shown in Figure 3.

**Figure 4:**
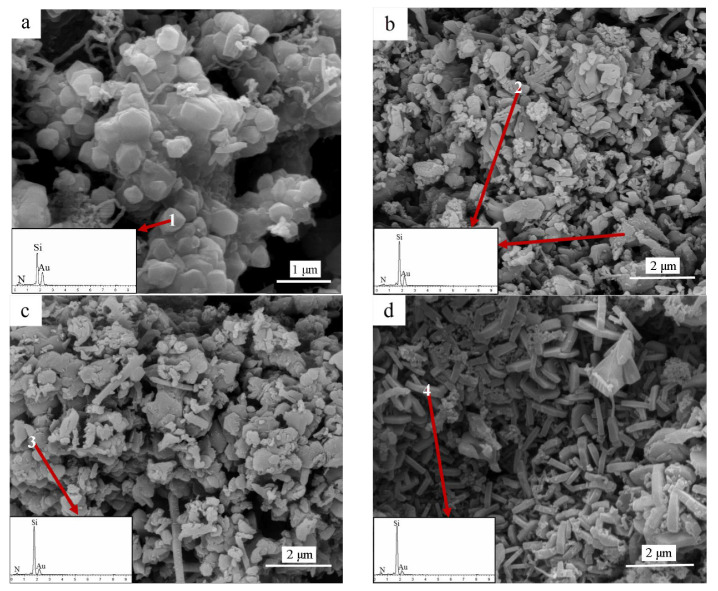
SEM images and EDS analysis (insets) of final products obtained by nitridation at 1250 °C for 5 h, using various catalysts. (**a**) catalyst-free, (**b**) Fe catalyst, (**c**) Co catalyst, (**d**) Ni catalyst. The red arrows and the numbers indicate the location of the analysis.

The detailed microstructure and elemental composition of the as-prepared Si_3_N_4_ samples were examined by FESEM and EDS analysis (insets in Figure 4 and Table 1). SEM images of the samples containing 2 wt% Fe, Co, and Ni are shown in Figure 4. The nitridation product mainly consists of irregularly elongated particles, equiaxed flakes, and short rod-like and hexagonal plate-like grains. As shown in Figure 4a, the catalyst-free sample contains many equiaxial grains with diameters in the micron range, and then numerous grains aggregate to form large particles. Spectra obtained from EDS analysis, given as the inset in Figure 4a, confirm the presence of only N and Si in the granular particles. As seen in Figure 4b, a number of anomalous elongated grains are observed for the samples obtained using the Fe catalyst. Coarse granular agglomerates, composed of α-Si_3_N_4_ particles, are randomly distributed in the samples, as shown in Figure 4b. The SEM image in Figure 4c reveals that coarse equiaxial flakes and glossy rod-like grains form agglomerates on the surface of the sample obtained using Co powder as the catalyst, similar to our previous results [38]. On the other hand, the SEM image of the sample obtained using the Ni catalyst (Figure 4d) indicates the formation of well-developed hexagonal plate-like α-Si_3_N_4_ grains with the thicknesses of several dozen nanometers, and these grains are randomly distributed in the sample. EDS results (insets in Figure 4b–d and Table 1) show that the anomalous elongated grains, flake-like and rod-like particles, and hexagonal plates consist of Si and N; the elemental amounts correspond to the Si_3_N_4_ phase, in agreement with the XRD and IR results.

**Figure 5:**
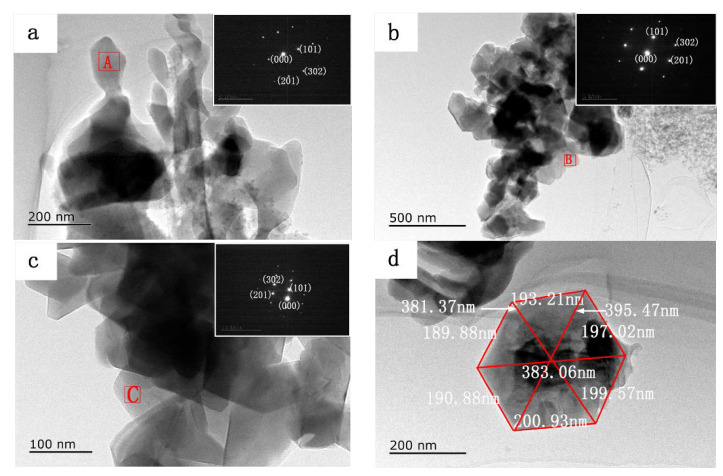
TEM images and SAED patterns (insets, area of A, B, and C) of samples containing obtained using catalysts. (**a**) 2 wt% Fe, (**b**) 2 wt% Co, (**c**) 2 wt% Ni, (**d**) an individual α-Si_3_N_4_ hexagonal plate in the sample obtained using Ni as the catalyst.

TEM observations along with SAED analysis were further carried out to identify the crystalline structure of the Si_3_N_4_ samples generated at 1250 °C. Figure 5 shows the transmission electron microscopy and SAED images of the samples obtained with Fe, Co, and Ni as catalysts. The TEM image of the sample obtained with Fe as the catalyst (Figure 5a) shows that elongated and granular grains agglomerate to form larger granules. The equiaxial α-Si_3_N_4_ flakes aggregate together to form large particles in the product obtained with Co as the catalyst (Figure 5b), as seen in the corresponding SEM image. The TEM image of the sample obtained with Ni as the catalyst (Figure 5c) shows hexagonal Si_3_N_4_ plates. The related SAED patterns (insets in Figure 5a–c) confirm that the anomalous elongated grains, flake-like and rod-like particles, and hexagonal plates comprise perfect single-crystalline α-Si_3_N_4_, wherein the diffraction spots correspond to the (201) and (101) lattice planes of α-Si_3_N_4_ (ICDD card No. 074749), in agreement with the SEM analysis. Figure 5d presents a high-magnification TEM image of the sample, showing a hexagonal plate with edge lengths of about 193 nm, 190 nm, 191 nm, 201 nm, 200 nm, and 197 nm with an α-Si_3_N_4_ crystal structure. Combined with the SEM results, an independent α-Si_3_N_4_ hexagonal plate with a diameter size of about 395 nm and edge length of about 197 nm can clearly be seen.

**Figure 6:**
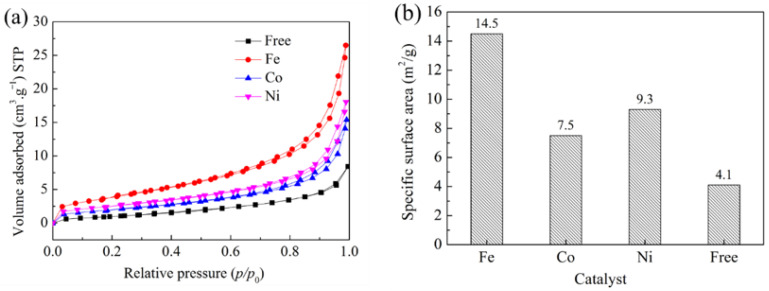
Nitrogen adsorption–desorption isotherms and specific surface area of samples containing different catalysts heated at 1250 °C for 5 h in NaCl–NaF molten salt media. (**a**) nitrogen adsorption–desorption isotherms and (**b**) specific surface area.

Figure 6a shows nitrogen adsorption–desorption isotherms of samples containing different catalysts heated at 1250 °C for 5 h in NaCl–NaF molten salt media. The nitrogen adsorption capacity of samples increased sharply, and the adsorption curve was biased towards the *y* axis at the low pressure area (P/P_0_ = 0.0–0.1), indicating that there were stable micropores in the samples. Moreover, the adsorption capacity increased slowly at P/P_0_ = 0.3–0.8 and increased significantly at P/P_0_ = 0.9–1.0. It can be confirmed that the adsorption–desorption isotherms of samples are typical type II, according to the IUPAC classification [45]. It is worth noting that the adsorption isotherms of samples, through the addition of catalysts, had a long lagged loop at a high relative pressure area, indicating that there were many mesopores in the samples. On the other hand, the adsorption capacity of sample with Fe catalyst was larger than that of the other two samples. However, the adsorption isotherm of catalyst-free sample has no hysteresis loop. Figure 6b shows the specific surface area of samples without catalyst or by addition of Fe, Co, and Ni powder as catalyst. As shown in Figure 6b, the specific surface area of the sample without catalyst was 4.1 m^2^/g. In contrast, the specific surface area of samples with Fe, Co, and Ni powder was obviously improved. When Fe powder is used as a catalyst, the specific surface area of the sample reaches 14.5 m^2^/g.

**Figure 7:**
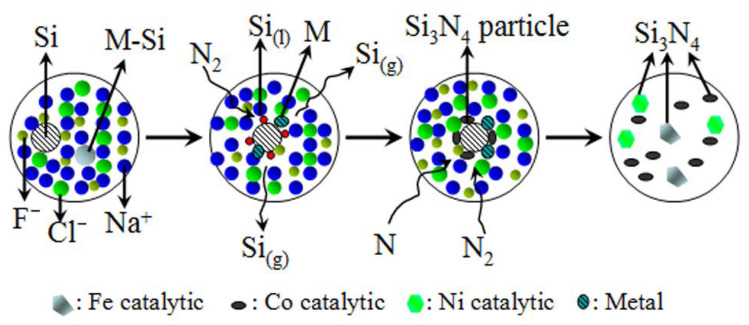
Suggested mechanism for the catalytic nitridation of Si powder.

During Si_3_N_4_ synthesis in molten salt system, silicon powder is slightly soluble in the molten salt system under high temperature conditions, and the dissolved silicon exists in the form of atoms or atomic groups. As the temperature increases, the surface tension and viscosity of the molten salt decrease significantly, and the dissolution and diffusion of silicon powder in the molten salt intensifies [22,39]. The introduction of catalysts has a crucial effect on the composition and microstructure of Si_3_N_4_ powder in the NaCl–NaF molten salt media. It was worth noting that the crystal shape of α-Si_3_N_4_ could be converted to anomalously elongated, granular, or hexagonal plate-like form by the addition of Fe, Co, or Ni. When metal catalysts are present in the samples, the mechanism of Si powder nitridation in the samples is that M-Si (M = Fe, Co and Ni) liquid phase was formed by reactions between metal (Fe, Co or Ni) and Si at low temperature. Moreover, the nucleation and growth of Si_3_N_4_ were controlled by different reactions in the molten salt media. Firstly, Si_3_N_4_ nucleation was mainly controlled by gas–solid reaction between dissolved Si atom and N_2_. While the growth of Si_3_N_4_ grain was established to be reaction between Si(g)/Si(l) and N/N_2_ in the system. However, there began to appear liquid phase in the samples containing Fe, Co, and Ni catalysts at different temperature. Therefore, amount of Si(g)/Si(l) in the system varied with catalyst type at nitridation temperature, which also marked Si_3_N_4_ crystal preferential growth in different directions. A mechanism for the catalytic of nitridation of Si powder is shown in Figure 7.

## 4. Conclusions

In summary, to meet the high-quality powder requirements for the preparation of ceramic substrates with low grain boundary size defects, a high α form Si_3_N_4_ powder with multiple morphologies was obtained through the nitridation of Si in molten state via the reaction of Si powder mixed with Na salts under N_2_ atmosphere in the presence of various transition metal catalysts. The nitridation of Si powder with 2 wt% catalysts (Fe, Co, or Ni) was carried out at 1250 °C for 5 h. While only 52.4 wt% α-Si_3_N_4_ was present in the catalyst-free sample, there were about 96 wt% α-Si_3_N_4_ and 4 wt% β-Si_3_N_4_ in the samples obtained using 2 wt% Ni, Fe, and Co, under the same reaction conditions. The addition of Fe, Co, and Ni catalysts not only promoted the formation of α-Si_3_N_4_ but also influenced the morphology of α-Si_3_N_4_ particles. When using Fe as the catalyst, several anomalously elongated α-Si_3_N_4_ grains were obtained, and randomly distributed in the sample. When using Co and Ni as the catalysts, equiaxial flake-like and rod-like α-Si_3_N_4_ particles, and hexagonal plate-like α-Si_3_N_4_ particles were obtained, respectively. The growth mechanism of α-Si_3_N_4_ was confirmed, which was mainly a vapor–solid or vapor–liquid–solid pattern.

## Figures and Tables

**Table 1:** Microarea percent ratio of points 1, 2, 3, and 4 in Figure 4/Atomic%.

Element	N	Si	Au
1	40.25	50.45	9.30
2	48.40	44.65	6.96
3	37.63	55.56	6.81
4	51.55	41.62	6.83

## Data Availability

Data available on request due to privacy restrictions. The data presented in this study are available on request from the corresponding author.
